# Achieving the health and well-being Sustainable Development Goals among adolescent mothers and their children in South Africa: Cross-sectional analyses of a community-based mixed HIV-status cohort

**DOI:** 10.1371/journal.pone.0278163

**Published:** 2022-12-08

**Authors:** Elona Toska, Wylene Saal, Jenny Chen Charles, Camille Wittesaele, Nontokozo Langwenya, Janina Jochim, Kathryn J. Steventon Roberts, Jason Anquandah, Boladé Hamed Banougnin, Christina Laurenzi, Lorraine Sherr, Lucie Cluver

**Affiliations:** 1 Centre for Social Science Research, University of Cape Town, Cape Town, South Africa; 2 Department of Sociology, University of Cape Town, Cape Town, South Africa; 3 Department of Social Policy and Intervention, University of Oxford, Oxford, United Kingdom; 4 Department of Infectious Disease Epidemiology, London School of Hygiene and Tropical Medicine, London, United Kingdom; 5 Institute for Global Health, University College London, London, United Kingdom; 6 Department of Statistics, University of Leeds, Leeds, United Kingdom; 7 Institute for Life Course Health Research, Stellenbosch University, Stellenbosch, South Africa; 8 Department of Psychiatry and Mental Health, University of Cape Town, Cape Town, South Africa; Medical Research Council, SOUTH AFRICA

## Abstract

The Sustainable Development Goals (SDGs) are a visionary and multi-sectoral agenda for human development. With less than a decade left to reach these targets, it is important to identify those at greatest risk of not meeting these ambitious targets. Adolescent mothers and their children are a highly vulnerable group. We mapped 35 SGD-related targets among 1,046 adolescent mothers and their oldest child (n = 1046). Questionnaires using validated scales were completed by 10- to 24-year-old adolescent girls and young women who had their first child before age 20 in an HIV-endemic district in the Eastern Cape province of South Africa. Maternal outcomes included 26 SDG-aligned indicators, while child-related outcomes included 9 indicators. Data was collected by trained researchers, following informed voluntary consent by the adolescent mothers and their caregivers. Frequencies and chi-square tests were conducted to compare progress along SDG-aligned indicators among adolescent mothers by HIV status. Overall, adolescent mothers reported low attainment of SDG-aligned indicators. While four in five adolescent mothers lived in poor households, nearly 93% accessed at least one social cash transfer and 80% accessed a child support grant for their children. Food security rates among adolescent mothers (71%) were lower than among their children (91%). Only two-thirds of adolescent mothers returned to school after childbirth, and only one-fifth were either studying or employed. Over half of adolescent mothers had experienced at least one type of violence (domestic, sexual or community). HIV-positive status was associated with higher rates of hunger and substance use, poorer school attendance, and higher rates of exposure to violence. Understanding progress and gaps in meeting the SDGs among highly vulnerable groups is critical, particularly for adolescent mothers and their children. These complex vulnerabilities suggest that programming for adolescent mothers must address their unique needs.

## Introduction

The Sustainable Development Goals (SDGs) represent a shared multi-sectoral vision for human development by 2030. With just under a decade left to drive progress towards the SDGs, it is critical to understand who is at greatest risk of not meeting the ambitious seventeen goals and 169 targets. As the COVID-19 pandemic has exacerbated existing vulnerabilities and resource inequalities globally, this imperative has become particularly important [1]. While few SDG indicators are adolescent-specific, we cannot meet these visionary goals without protecting and promoting adolescent development, especially for at-risk groups such as adolescent mothers. Recent data suggests that pregnancy and fertility-related complications, alongside HIV, are the lead causes of mortality among young adolescent women ages 15–19 [2].

The limited data available on adolescent mothers and their children in resource-constrained settings suggests that they are a highly vulnerable group. Adolescent parenthood is a marker of disadvantage, linked to child developmental delay [3–5], and frequently preceded and accompanied by severe adversity [6–9]. Predictors of adolescent parenthood include orphanhood [10], abuse, neglect [11], and forced sex [12]. Socioeconomic drivers of early parenthood include school non-enrolment [13], poverty and low future aspirations [14]. Children born to adolescent mothers in low- and middle-income countries are likely to have poor child health outcomes [4, 15]: they are more likely to be born underweight [4] and/or preterm, and have increased risk of mortality [16–18]. These children also tend to have lower cognitive development, poorer nutritional status and uneven school progression [19]. These risk factors link to lifelong adverse health outcomes for children born to young mothers, possibly setting up cycles of intergenerational disadvantage [20]. Less is known about the long-term effects of adolescent motherhood on their children, particularly in HIV-endemic communities, beyond their early health outcomes. Although there is growing interest in how we can best support adolescent mothers affected by HIV, a recent review did not find any studies documenting the effect of HIV on outcomes of adolescent mothers and their children [21].

Sub-Saharan Africa will be home to more than 16.4 million adolescent mothers by 2030 [22]. However, only a handful of studies have documented impacts of adolescent motherhood beyond the health of adolescent mothers and their children, with studies on non-health outcomes primarily using qualitative data or small sample sizes [23–25]. No studies have examined adolescent mother and child outcomes simultaneously within the holistic human development-centred lens of the SDGs [21]. In the current environment, the massive shocks to livelihoods, food security and economic opportunities due to COVID-19 are likely to result in higher rates of sexual risk-taking and higher prevalence of gender-based violence experienced by adolescents and young people [26, 27]. Combined with disruptions in healthcare access, particularly family planning and contraception, the structural impacts of COVID-19 are already resulting in a higher number of unintended pregnancies among adolescents and young women in some countries [28].

To design evidence-based responses, it is essential to understand which SDGs adolescent mothers and their children are failing to reach. This study had two interlinked objectives: first, to investigate how adolescent mothers and their children in South Africa are faring with regards to key human development SDG-aligned indicators, and second, to investigate the effect of HIV on reaching these goals.

## Methods

### Study design

In 2018–2019, we interviewed n = 1,046 10- to 24-year-old adolescent girls and young women who had their first child before the age of 20. The sample included n = 311 adolescent mothers living with HIV and n = 735 not living with HIV. We also collected data from their oldest child (n = 1,046).

### Setting and sample

This study was conducted in the Eastern Cape province, South Africa. The study was located in a mixed rural-urban district with the highest HIV prevalence rate of 38.8% among women in antenatal care in the Eastern Cape [29]. The study included 10- to 24-year-old adolescent girls and young women who had their first child before the age of 20. To minimise recruitment bias, we identified eligible participants through a comprehensive set of sampling strategies that included healthcare facilities and community-based approaches [30]. The research team co-developed six recruitment channels with local experts and an advisory group of adolescent mothers, including: 1) all known district health facilities (n = 73), 2) maternity obstetric units (n = 9), 3) a randomly selected subsamples of secondary schools (n = 43), 4) neighbouring adolescents of eligible consenting participants to reduce unintended stigma, 5) referrals by social workers and service providers, and 6) community referrals by adolescent mothers themselves. Refusals and successful recruitment rates were recorded for each channel: 95%-98% of all eligible mothers identified through each channel were successfully enrolled in the study.

### Data collection procedures

Voluntary informed consent was obtained from adolescents and their caregivers where adolescents were under 18, following international and national guidelines for consent among vulnerable populations. In instances where the adolescent parent was not the main caregiver of the child(ren), additional consent was obtained from the primary caregiver of the infant, as identified by the adolescent parent. Data collection tools were piloted with n = 25 adolescents living with HIV and n = 9 adolescent mothers. Pilot data collection tools included 1) self-reported adolescent interviews focusing on adolescent lives and pregnancy/parenting experiences, 2) self-reported child data from the primary caregiver (either the adolescent mother or another family member), 3) standardised child development measures, 4) child medical records extracted from the South African government’s Road to Health booklets, and 5) HIV patient file and maternity case records for adolescent mothers. Questionnaires were administered through tablets using OpenDataKit, using skip patterns to reduce questionnaire burden, and strict data quality controls to minimise errors and missing data. Data were stored in protected servers at the Universities of Oxford and Cape Town.

Questionnaires and consent forms were translated from English into isiXhosa, the predominant local language, and were back-translated using standard procedures to ensure accuracy [31]. Interviewers received extensive training in conducting research with vulnerable children and families, safeguarding and referral pathways if needed. Questionnaires were tablet‐based, allowing adolescents to answer sensitive questions privately and participate in the language of their choice. No incentives were provided, but all participants received a snack and a small pack with toiletries, toys, and stationery for themselves and their child(ren). All participants received a certificate acknowledging participation.

Measures to maintain confidentiality included allowing participants to select the interview location; assigning a unique serial number to each participant; removing identifiers from questionnaires; and storing field notes and consent forms in password-controlled electronic files and locked cabinets. Where participants reported recent abuse, rape or risk of significant harm, referrals were made to child protection and health services, with follow-up support provided. Ethical approvals were obtained from the Universities of Oxford (R48876/RE001-3, SSD/CUREC2/12–21) and Cape Town (HREC 226/2017, CSSR 2013/4), Eastern Cape Departments of Health and Basic Education, and participating health and educational facilities.

Adolescent mothers advised the study team on recruitment methods, which were incorporated in the study protocol. The questionnaire was piloted and tested with adolescent advisors from our Teen Advisory Groups in two South African provinces and the adolescent mother advisors. Feedback helped refine the questionnaire content and approach prior to administering the questionnaire. Community leaders were engaged to ensure recruitment in communities recruitment strategies were sensitive and effective, whilst minimising the risk of stigma.

### Measures

We mapped data from adolescent mothers and their children against 35 human development SDG-aligned indicators (26 for mothers and 9 for children) within 8 human development SDGs (8 for mothers and 5 for their oldest children). SDG-aligned indicators were operationalised through variables as outlined in S1 Table. Sociodemographic characteristics included: age at interview, age at first child, age of child(ren), rural residence, informal housing, HIV status, and caregiving arrangements for both adolescent and their child(ren). Self-reported HIV status for adolescent mothers was confirmed by medical records.

### Data analysis

Data analysis was conducted using STATA 16. Frequencies were computed for each SDG-aligned indicator, followed by chi-square tests comparing adolescent mothers by HIV status. SDG-aligned indicators for children were reported for the entire sample and by maternal HIV status. Missing data levels are reported for each key variable.

## Results

Table 1 summarises the characteristics of the study sample by maternal HIV status. The average age of participating adolescent mothers was 18.3 years (SD 1.9) at the time of the interview, with 16.7 years (SD 1.7) as the mean age at birth of their first child. 29.7% (n = 311) of participants were confirmed to be living with HIV. One in twelve adolescent mothers had multiple births. Adolescent mothers living with HIV were, on average, 2 years older than their uninfected peers, and their children were older (18 months versus 12 months old on average). Over 96% of adolescent mothers were the primary caregiver to their children, and 99% of them lived with their own caregiver, highlighting the intergenerational composition of the households where adolescent mothers and their children live. Nearly three-quarters of participants lived in urban communities, with one-fifth living in informal housing.

**Table 1:** Baseline socio-demographics.

Socio-demographic factors	Adolescent mother not living with HIV (n = 735)	Adolescent mother living with HIV (n = 311)	All (n = 1046)	p-value
Current age in years [range 12–25] (mean, SD)	17.7 (1.5)	19.7 (1.9)	18.3 (1.9)	≤0.001
≤13	3(0.4%)	0	3 (0.29%)	0.26
14–16	142 (19.3%)	19 (6.1%)	161 (15.4%)	≤0.001
17–19	545 (74.2%)	131 (42.1%)	676 (64.3%)	≤0.001
≥ 20	45 (6.1%)	161 (51.8%)	206 (19.7%)	≤0.001
Age at first child (mean, SD) [range 11–23]	16.4 (1.4)	17.6 (2.0)	16.7 (1.7)	≤0.001
Current average oldest child age (months, range 0–108)	17.3 (15.7)	28.2 (23.8)	20.5 (19.1)	≤0.001
Number of children–total (n)	772	373	1,145	
One child	698 (95.0%)	253 (81.4%)	951 (90.9%)	≤0.001
Two children	37 (5.0%)	54 (17.4%)	91 (8.7%)	
Three children	0 (0%)	4 (1.3%)	4 (0.4%)	
Adolescent mother is primary caregiver to oldest child	703 (95.7%)	295 (94.9%)	999 (95.5%)	0.738
*(Missing value n = 1)*				
Adolescent mother lives with their caregiver at home	730 (99.3%)	305 (98.1%)	1035 (99.0%)	0.07
Urban	512 (69.7%)	235 (75.6%)	747 (71.4%)	0.05
Informal housing	152 (21.0%)	73 (24.6%)	225 (22.0%)	0.209
*(Missing values n = 25)*				

### SDG attainment among adolescent mothers and children (Table 2)

**Table 2:** SDGs-aligned outcomes among adolescent mothers and their children.

	Adolescent mothers not living with HIV (n = 735)	Adolescent mothers living with HIV (n = 311)	Total (n = 1046)	p-value
**SDG 1: No poverty**				
*No household poverty*				
Access to 7 basic household needs	154 (21.0%)	57 (18.3%)	211 (20.2%)	0.33
Basic necessities for oldest child	53 (7.2%)	39 (12.5%)	92 (8.8%)	0.014
*(Missing values n = 1)*				
Employment at home	420 (57.1%)	184 (59.2%)	604 (57.7%)	0.545
*Access to social protection*				
Access to any government welfare grant	**670 (91.2%)**	**299 (96.1%)**	**969 (92.6%)**	**0.01**
Access to child support grant	**571 (77.7%)**	**263 (84.6%)**	**834 (79.7%)**	**0.01**
*(Missing values n = 12)*				
Access to food gardens and parcels	121 (16.5%)	44 (14.2%)	165 (15.8%)	0.35
**SDG 2: Zero Hunger**	**Adolescent mothers not living with HIV (n = 735)**	**Adolescent mothers living with HIV (n = 311)**	**Total (n = 1046)**	**p-value**
Adolescent mother food security (past-week)	533 (72.5%)	214 (68.8%)	747 (71.4%)	0.225
Child food security (past-week)	**627 (85.3%)**	**253 (81.4%)**	**880 (84.1%)**	**0.01**
*(Missing values n = 1)*				
Breastfeeding exclusively in the first six months	**153 (20.8%)**	**119 (38.3%)**	**272 (26.0%)**	**≤0.001**
*(Missing values n = 1)*				
Good nutrition for child	583 (79.3)	249(80.1%)	832 (79.5%)	0.526
*(Missing values n = 1)*				
**SDG 3: Good Health and Well-being**	**Adolescent mothers not living with HIV (n = 735)**	**Adolescent mothers living with HIV (n = 311)**	**Total (n = 1046)**	**p-value**
Access to antenatal care services	156 (21.2%)	80 (25.7%)	236 (22.6%)	0.112
*(Missing values n = 1)*				
Facility-based birth	730 (99.3%)	309 (99.4%)	1039 (99.3%)	0.946
*(Missing values n = 1)*				
Clinic accessibility	558 (75.9%)	230 (74.0%)	788 (75.3%)	0.501
No mental health issues	**688 (93.6%)**	**280 (90.0%)**	**968 (92.5%)**	**0.04**
Enhanced mental health	**540 (73.5%)**	**207 (66.6%)**	**747 (71.4%)**	**0.024**
No substance abuse	**642 (87.4%)**	**237 (76.2%)**	**879 (84.03%)**	**≤0.001**
Child did not have waterborne/ communicable disease	594 (80.8%)	236 (75.9%)	830 (79.4%)	0.072
**SDG 4: Quality Education**	**Adolescent mothers not living with HIV (n = 735)**	**Adolescent mothers living with HIV (n = 311)**	**Total (n = 1046)**	**p-value**
*Highest education level*				
Primary or secondary school	**429 (58.4%)**	**63 (20.3%)**	**492 (47.0%)**	**≤0.001**
University or college	41 (5.6%)	22 (7.1%)	63 (6.0%)	0.353
School enrolment	**470 (64.0%)**	**85 (27.3%)**	**555 (53.1%)**	**≤0.001**
*School access & pregnancy*				
Enrolled before pregnancy	**691 (94.5%)**	**231 (76.7%)**	**922 (89.3%)**	**≤0.001**
*(Missing values n = 14)*	4 (0.5%)	10 (3.2%)	12 (1.1%)	
Attended school during pregnancy	**539 (78.0%)**	**144 (62.3%)**	**683 (74.1%)**	**≤0.001**
Dropped out before pregnancy	**40 (5.4%)**	**70 (22.5%)**	**124 (11.9%)**	**≤0.001**
Returned to school after pregnancy	**526 (74.6%)**	**142 (51.2%)**	**668 (68.0%)**	**≤0.001**
*(Missing values n = 12)*				
Grade for age progression	**289(39.3%)**	**69 (22.2%)**	**358 (34.2%)**	**≤0.001**
Child attending ECD (for children between 1–5 years old)	167 (22.7%)	87 (28.0%)	254 (24.3%)	0.172
(*Missing values n = 70)*	52 (7.1%)	18 (5.8%)		
**Child cognitive development (Mullen standardised composite score [49–155])**	**94.1**	**91.1**	**93.2**	**0.03**
**SDG 5: Gender Equality**	**Adolescent mothers not living with HIV (n = 735)**	**Adolescent mothers living with HIV (n = 311)**	**Total (n = 1046)**	**p-value**
No high-risk sex	84 (11.4%)	28 (9.0%)	112 (10.7%)	0.246
*Access to sexual and reproductive health–contraception use*				
Hormonal contraception	466 (63.4%)	199 (64.0%)	665 (63.6%)	0.857
Condom use at last sex	188 (25.6%)	92 (29.6%)	280 (26.8%)	0.181
Dual protection	136 (18.5%)	65 (20.9%)	201 (19.2%)	0.368
Father spent time with child *(Missing values n = 1)*	67 (9.12%)	30 (9.7%)	97 (9.3%)	0.547
Childcare support (Formal & Informal)	517 (70.3%)	201 (64.6%)	718 (68.6%)	0.069
**SDG 8: Decent Work and Economic Growth**	**Adolescent mothers not living with HIV (n = 735)**	**Adolescent mothers living with HIV (n = 311)**	**Total (n = 1046)**	**p-value**
In education or employed	**475 (64.6%)**	**94 (30.2%)**	**569 (54.4%)**	**≤0.001**
Employment readiness	**93 (12.7%)**	**72 (23.2%)**	**165 (15.8%)**	**≤0.001**
**SDG 9: Building resilient infrastructure, promote inclusive and sustainable industrialization and foster innovation**	**Adolescent mothers not living with HIV (n = 735)**	**Adolescent mothers living with HIV (n = 311)**	**Total (n = 1046)**	**p-value**
Mobile phone access (ownership)	591 (80.4%)	239 (76.9%)	830 (79.4%)	0.194
**SDG 16: Peace, Justice and Strong Institutions**	**Adolescent mothers not living with HIV (n = 735)**	**Adolescent mothers living with HIV (n = 311)**	**Total (n = 1046)**	**p-value**
No exposure to violence at home (physical, emotional nor domestic)	634 (86.3%)	260 (83.6%)	894 (85.5%)	0.265
No sexual/ relationship-related violence	**509 (69.3%)**	**177 (56.9%)**	**686 (65.6%)**	**≤0.001**
No exposure to community violence	530 (72.1%)	236 (75.9%)	766 (73.2%)	0.207
No types of violence	**338 (46.0%)**	**118 (37.9%)**	**456 (43.6%)**	**0.016**
Child has full documentation available to access services (*Missing value n = 1)*	670 (91.2%)	284 (91.3%)	954 (91.2%)	0.933

#### Goal 1 –No poverty

Adolescent mothers in the study reported high levels of poverty–only one in five (20%) had access to seven basic needs at home. An even lower proportion of adolescent mothers (15.3%) were able to afford basic child-related supplies like baby nappies and formula. Over 90% of adolescent mothers lived in households that received at least one government welfare grant. Almost four in five adolescent mothers accessed the child support grant (CSG). Access to social cash transfers was higher in households where adolescent mothers living with HIV resided, and adolescent mothers living with HIV were more likely to access the child support grant for their children (84.6% vs 77.7%, p = 0.01). Overall, adolescent mothers–independent of HIV status–lived in resource-constrained communities, with over half of households having at least one person employed (57.7%).

#### Goal 2 –Zero hunger

Nearly a quarter of adolescent mothers reported not having enough food at home in the prior week, which did not differ by HIV status. One in six adolescent mothers received food support by accessing food gardens or parcels. Exclusive breastfeeding rates were low overall (26.0%) but adolescent mothers living with HIV were more likely to report exclusive breastfeeding for the first six months of their children’s lives (38.3% vs 20.8%, p<0.001). Nine in ten children had enough food in the past week, with rates of food security lower among adolescent mothers living with HIV. 79.5% of all children had access to regular fruit and vegetables as part of their diet.

#### Goal 3 –Good health

About one in four adolescent mothers accessed more than five antenatal appointments and 99% gave birth at a health facility, with no differences by HIV status. More than nine in ten (93%) adolescent mothers reported no mental health issues with just over 70% reporting enhanced mental health, i.e., no common mental health disorders and high future aspirations. Adolescent mothers living with HIV were less likely to report positive mental health (90.0% vs 93.6%, p = 0.04). One in six adolescent mothers reported substance use, with higher rates among adolescent mothers not living with HIV (87.4% vs 76.2%, p<0.001). Four in five children were healthy with no recent symptoms of water-borne disease and other communicable diseases, with no differences by the HIV status of their mother.

#### Goal 4 –Quality education

Although nine in ten (89.3%) adolescent girls and young women were enrolled in school before they became pregnant, only three-quarters (74.1%) attended school during pregnancy and under two-thirds (68.0%) returned to school after giving birth to their child. Adolescent mothers living with HIV were less likely to report positive education outcomes: they were less likely to have been in school before they became pregnant (76.6% vs 94.5%, p<0.001), less likely to attend school during pregnancy (62.3% vs 78.0%, p<0.001), and less likely to return to school after giving birth (51.2% vs 74.65, p<0.001). Two-thirds of adolescent mothers reported delayed grade progression, and adolescent mothers living with HIV were more likely to report educational delays (22.2% vs 39.3%, p<0.001). More than one-quarter (28.0%) of the children of adolescent mothers attended early childhood development services, with no difference by maternal HIV status. Children of adolescent mothers living with HIV were more likely to score lower on the Mullen Scales of Early Learning compared to children of mothers not living with HIV (91.1 vs 94.1, p<0.03).

#### Goal 5 –Gender equality

Nine out of ten adolescent mothers engaged in high-risk sex, with no difference by HIV status. Nearly two thirds of adolescent mothers reported hormonal contraception use, but just over one-quarter reported condom use at last sex, with no difference by HIV status. Only a tenth of fathers spent time with their children, and seven in ten adolescent mothers received childcare support. Both did not differ by HIV status.

#### Goal 8 –Decent work and economic growth

54% of adolescent mothers were either employed or enrolled in school at the time of the interview, with adolescent mothers living with HIV less likely to be in school or employed (30.3% vs 64.4%, p<0.001). Only one in six reported elements of employment readiness (had a CV, reference letter, employment information via mobile phones and job-finding support through their social networks) with adolescent mothers living with HIV reporting higher rates of employment readiness (23.3% vs 12.7%, p<0.001).

#### Goal 9 –Resilient infrastructure

Four in five adolescent mothers owned a phone, with no differences by HIV status.

#### Goal 16 –Peace, justice and strong institutions

86% of adolescent mothers reported no exposure to physical, emotional or domestic abuse at home, and 73% of adolescent mothers reported no exposure to community violence. There was no difference by HIV status for both violence at home and in the community. One-third of adolescent mothers reported exposure to sexual/relationship-related violence; a significantly higher proportion of adolescent mothers not living with HIV reported no exposure to sexual/relationship violence (69.3% vs 56.9%, p<0.001). Overall, three of five reported exposure to all types of violence, with adolescent mothers not living with HIV reporting lower rates of violence. 92% of children of adolescent mothers had access to birth registration and Road to Health documents–two key facilitators of accessing support services and systems in South Africa–and this did not differ by the HIV status of their mothers.

## Discussion

Examining the progress of adolescent mothers and their children through the holistic lens of the Sustainable Development Goals, we highlighted severe gaps across multiple goals and targets. In this study, we report the first known findings on the progress made by adolescent mothers and their children, living in high HIV-prevalence resource-constrained communities, in attaining of a wide range of human development indicators. Results highlight significant gaps in terms of outcomes across several SDGs, including poverty reduction, food security, education, health and violence prevention. Adolescent mothers in South Africa had high rates of facility-based births but low rates of access to antenatal care [32]. Additional research on the HIV-related outcomes of adolescent mothers living with HIV is needed. Adolescent mothers living with HIV also reported worse mental health, particularly when considering their future aspirations and self-efficacy, than their HIV-uninfected peers. A recent systematic review found only a handful of qualitative studies that shed light on the mental health experiences of adolescent mothers [33] with recent quantitative analyses highlighting the complex patterns of common mental health disorders among this highly vulnerable group [34].

While adolescent mothers lived in food insecure households, the rates of access to food and nutrition among their children were higher. These findings confirm qualitative findings that even though the majority of adolescent mothers were not planning early pregnancy, they were dedicated to being the best mothers that they could be, prioritising the growth and development of their child [35]. Adolescent mothers living with HIV reported lower rates of child food security but higher rates of exclusive breastfeeding in the first six months of their child’s life. Breastfeeding support should target and provide responsive services to two-thirds of adolescent mothers living with HIV who are not exclusively breastfeeding for their children’s first six months. Special attention may be needed for breastfeeding adolescent mothers who return to school, particularly given emerging evidence on early return to school among adolescent mothers in this study [36].

Our findings highlight important missed opportunities. Although education is globally recognised as a social vaccine [37], adolescent mothers fall behind both during pregnancy and after birth [38]. It is notable that gender equality is very far from being reached: adolescent mothers are largely un-supported by partners or fathers of children. While two thirds of fathers made some financial contributions towards children, they remain largely uninvolved in childcare and childrearing [39]. As a result, a large proportion of adolescent mothers struggled to afford necessities for their children, such as nappies and formula [40].

Half of the adolescent mothers were neither enrolled in school nor employed–much lower than national enrolment rates for females in this age group in South Africa in 2018 [41]. Low rates of school completion or employment–a marker of socioeconomic development among older adolescent mothers–fuel intergenerational cycles of deprivation which most likely resulted in adolescent motherhood. A recent analyses of a combined dataset from two provinces in South Africa found similarly low rates of return to school following childbirth among adolescent mothers [36]. Although access to mobile phones was relatively high, use for health information or employment opportunities was extremely low. Given the importance of accessing such information to bridge access to healthcare services and employment support, opportunities to capitalize on mobile phone programmes among adolescent mothers should be explored.

Although all adolescent mothers experienced low rates of SDG achievement, living with HIV appears to be linked to worse SDG progress across multiple indicators. However, these mothers were also more likely to report exclusive breastfeeding for at least six months (although rates of exclusive breastfeeding overall were low), access to social protection (a government child support grant), and employment readiness. Adolescent mothers living with HIV were more likely to be older, which may explain some of these differences. Additional analyses to account sociodemographic differences would be important to quantify the potential syndemic relationship between HIV and adolescent motherhood on multi-dimensional outcomes of adolescent mothers and their children, including breastfeeding, access to schooling and employment. Understanding the impact of HIV on different SDG-related outcomes is critical to tailoring interventions to the needs of adolescent mothers [21].

Children of adolescent mothers are growing up in conditions that put their future attainment of SDGs at risk [23]. Access to provisions such as childcare support and early childhood development is very limited [42], with missed opportunities both to provide stimulation for children and allow mothers to return to education or employment [43, 44]. In the context of potential detrimental effects due to HIV or ART exposure in utero and post-partum on child development [45], understanding patterns, barriers and facilitators of childcare access is an important step to designing supportive services for adolescent mothers and their children.

### Limitations

Our study has some limitations: first, the data is cross-sectional, and analyses are descriptive, and future research could valuably examine trends over time. Moreover, adolescent mothers living with HIV included in the study were more likely to be older, therefore additional analyses is needed to disentangle the effect of HIV and motherhood on achievement across different life domains. Second, all data is self-reported, except for maternal HIV status, which was verified through medical records where available, with appropriate ethical approvals. The study was conducted in South Africa, so the results may not be generalizable. However, the district where it took place has similar HIV-prevalence rates, health systems and structural drivers to that of neighbouring countries in Southern Africa, such as the Kingdoms of Lesotho, Eswatini, and Botswana.

Despite these limitations, this data is the first snapshot from a unique and large cohort of adolescent mothers and their children–the largest from the lower- and middle-income countries. These initial findings respond to a growing interest in understanding adolescent mothers’ experiences beyond health, taking an intergenerational and life-course approach to improve the well-being of young people, their children and future generations. Interventions are needed to allow adolescent mothers to access existing provisions, and tailor new ones to disrupt vulnerability pathways. A recent review documented a series of small-scale promising practices to support adolescent mothers and their children focusing on HIV and health-related outcomes representing the first steps in building an evidence base [21]. However, there are clearly substantive gaps in the development, testing and delivery of services delivered to this vulnerable, multi-generational group. Programming and support must acknowledge their unique multi-dimensional needs, spanning beyond HIV and physical health.

## Supporting information

S1 Table Summary of human development indicators and measures for adolescent mothers and their children. (DOCX)

S1 Checklist Reporting checklist for cross sectional study. (DOCX)
