# Supplementary material for: Achieving the health and well-being Sustainable Development Goals among adolescent mothers and their children in South Africa: Cross-sectional analyses of a community-based mixed HIV-status cohort
Source: PLoS One. 2022 Dec 8;17(12):e0278163. doi: 10.1371/journal.pone.0278163 (PMC9731463; doi:10.1371/journal.pone.0278163)
Supplement: S1 Table — (DOCX) [file pone.0278163.s001.docx]

*Online Supplementary Table 1. Summary of human development indicators and measures for adolescent mothers and their children*

| ***SDG*** | ***SDG indicator*** | ***Adolescent mothers*** | | ***Children of adolescent mothers*** | |
| --- | --- | --- | --- | --- | --- |
|  |  | ***Operationalised SDG-aligned indicator*** | ***Coding/ Variable type*** | ***Operationalised SDG-aligned indicator*** | ***Coding/ Variable type*** |
| **1 – End poverty in all its forms everywhere** | 1.3.1 Proportion of population covered by social protection floors/systems | Access to government cash transfers and social protection | *Access to any government welfare grant (overall household for any eligible children)* | Child support grant for oldest child | *Adolescent mother received grant for at least one of her children* |
|  |  | Access to food gardens and parcels | *Adolescent participant received monthly parcels or have a food garden* |  |  |
|  | 1.4.1 Proportion of population living in households with access to basic services | No household poverty | *Access to 7 basic household needs using nationally representative SA Social Attitudes Survey, including affording school stationery, fees for doctors and medicines, and basic clothing. ^1^*  *Employment at home (someone at home is employed and a grant is received)* | Basic necessities for oldest child | *Adolescent mother can afford necessities, such as nappies, food and clothes at least once every 2 weeks for oldest child* |
| **2 – End hunger, achieve food security and improved nutrition and promote sustainable agriculture** | 2.1.2 Prevalence of moderate or severe food insecurity in the population | Adolescent mother food security (past-week) | *Sufficient food for adolescent parents in the home every day in the past week and three meals per day ^2^* | Child food security (past-week) | *Sufficient food for children in the home every day in the past week* |
|  | 2.1.1 Prevalence of undernourishment |  |  | Breastfeeding (exclusively) | *Participants exclusively breastfed their oldest child in the first 6 months* |
|  |  |  |  | Good nutrition for child | *Children (>6 months) were fed solids, and fruit or vegetables.* |
| **3 – Ensure healthy lives and promote well-being for all at all ages** | 3.8.1 Coverage of essential health services | Access to antenatal health services | *Adolescent mother’s attendance to five or more antenatal appointments* |  |  |
|  |  | Facility-based birth | *Adolescent mother gave birth to their oldest child at a health facility* |  |  |
|  | 3.b.1 Proportion of the population with access to affordable medicines and vaccines on a sustainable basis | Clinic accessibility | *The adolescent mother had enough money for transport to get to the clinic and it’s safe to go to the clinic* |  |  |
|  | 3.4.2 Suicide mortality rate | No mental health issues | *No common mental disorders* |  |  |
|  |  | Enhanced mental health | *Adolescent participant has no common mental health issues and they believe they will achieve their future goals (future job, happy relationships, good health, afford necessities, healthy children, have a house)* |  |  |
|  | 3.5.2 Harmful use of alcohol | No substance abuse | *No alcohol use within the past-month* |  |  |
|  | 3.3 Combat hepatitis, water-borne diseases and other communicable diseases |  |  | Child did not have waterborne disease (diarrhoea) and other communicable disease | *The oldest child did not have diarrhoea or runny tummy for more than 2 days and no other serious infectious diseases (Tuberculosis, meningitis, pneumonia, measles)* |
| **4 – Ensure inclusive and equitable quality education and promote lifelong learning opportunities for all** | 4.3.1 Participation rate of youth and adults in formal and non-formal education and training in the previous 12 months | Highest education level completed | *Primary school, high school, or University/College/other FET* |  |  |
|  |  | School enrolment  School access & pregnancy | *General school enrolment.*    *School enrolment for adolescent mothers before, during, and after pregnancy* |  |  |
|  | 4.6.1 Percentage of population in a given age group achieving at least a fixed level of proficiency in functional (a) literacy and (b) numeracy | Grade for age progression | *Correct grade for age progression/passed matric in time for adolescent mothers* |  |  |
|  | 4.2.2 Participation rate in organised learning (one year before official primary entry age) |  |  | Early Childhood Development attendance for child  Child cognitive development | *Attendance of oldest child at ECD (for children between 1-5 years)*  *Composite score of early learning for four developmental domains (fine motor skills, visual reception, expressive language, and receptive language) – measured using the Mullen Scales of Early Learning ^3, 4^* |
| **5 – Achieve gender equality and empower all women and girls** | 5.6.1 Proportion of women aged 15-49 years who make their own informed decisions regarding sexual relations, contraceptive use and reproductive health  5.6.1 Proportion of women aged 15-49 years who make their own informed decisions regarding sexual relations, contraceptive use and reproductive health | No high-risk sex | *No gifts were received for having sex, the current sexual partner was not older than the adolescent by at least 5 years, no sex on drugs, protected sex using a condom, no multiple partners* |  |  |
|  |  | Contraception use | *Hormonal contraception: adolescent mother self-reports use of oral contraceptives, injectables, or implant; Condom use at last sex: adolescent used condom for entire duration of the most recent sexual act; Dual protection: adolescent used both hormonal contraception and condom at last sex, and abstinent adolescents* |  |  |
|  |  | Father spends time with child | *Father of the child devotes time to raise the child* |  |  |
|  |  | Childcare support | *Adolescent mother receives combinations of all types of help in looking after the child and helping with childcare (non-financial)* |  |  |
| **8 – Promote sustained, inclusive and sustainable economic growth, full and productive employment and decent work for all** | 8.6.1 Proportion of youth (aged 15-24 years) not in education, employment or training | In education or employment  Employment readiness | *Adolescent is in education or employment*  *Adolescent mother has either a CV, a reference letter, access to information or CV/job employment support* |  |  |
| **9 – Build resilient infrastructure, promote inclusive and sustainable industrialization and foster innovation** | 9.c.1 Proportion of population covered by a mobile network, by technology | Mobile phone access (ownership) | *Adolescent mother has access to a basic phone or smart phone* |  |  |
| **16 – Promote peaceful and inclusive societies for sustainable development, provide access to justice for all and build effective, accountable and inclusive institutions at all levels** | 16.2.1 Proportion of children aged 1-19 years who experienced any physical punishment and/or psychological aggression by caregivers in the past month **AND** 16.2.3 Proportions of young women and men aged 18-29 years who experienced sexual violence by age 18 | No exposure to violence at home - physical or emotional abuse or domestic violence  No sexual/relationship-related violence | *Participant has not been exposed to any physical or emotional abuse in the past year or domestic violence*  *No gifts were received for having sex, the current sexual partner was not older than the adolescent by at least 5 years, no sexual abuse nor IPV* |  |  |

|  | 16.3.1 Proportion of victims of violence in the previous 12 months who reported their victimization to competent authorities or other officially recognised conflict resolution mechanisms  16.9.1 Proportion of children under 5 years of age whose births have been registered with a civil authority | No exposure to community violence  No exposure to all types of violence | *Adolescent participant did not experience an event of community violence*  *Adolescent mother did not experience exposure to all types of violence* |  |  |
| --- | --- | --- | --- | --- | --- |
|  |  |  |  | Full documentation to access services | *The oldest child has road to health card and birth registration available* |

1 Pillay U, Roberts B, Rule SP. South African Social Attitudes: Changing Times, Diverse Voices. Cape Town, South Africa: HSRC Press; 2006. 391 p.

2. Labadarios D, Steyn NP, Maunder EMW, MacIntryre U, Gericke G, Swart R, et al. The National Food Consumption Survey (NFCS): South Africa, 1999. Public Health Nutr. 2005;8(5):533–43.

3. Boivin MJ, Nakasujja N, Sikorskii A, Opoka RO, Giordani B. A randomized controlled trial to evaluate if computerized cognitive rehabilitation improves neurocognition in ugandan children with HIV. AIDS Res Hum Retroviruses. 2016 Aug;32(8):743–55.

4. Mullen EM. Mullen Scales of Early Learning. AGS Edition; 1995.
